# Re-Shaping the Pancreatic Cancer Tumor Microenvironment: A New Role for the Metastasis Suppressor NDRG1

**DOI:** 10.3390/cancers15102779

**Published:** 2023-05-16

**Authors:** Jiawei Chang, Zoe H. Y. Lo, Shafi Alenizi, Zaklina Kovacevic

**Affiliations:** 1 School of Medical Sciences, Faculty of Medicine & Health, University of Sydney, Sydney 2006, Australia; 2 Department of Physiology, School of Biomedical Sciences, Faculty of Medicine & Health, University of NSW, Sydney 2052, Australia

**Keywords:** cancer, pancreatic cancer, pancreatic ductal adenocarcinoma, pancreatic cancer tumor microenvironment, NDRG1, extracellular vesicles, metabolism

## Abstract

**Simple Summary:**

Pancreatic cancer remains the leading cause of cancer death globally. With no reliable early detection strategy and limited treatment options, up to 90% of patients will lose their lives to this disease. In this review, we bring together recent advances in understanding the pancreatic cancer tumor microenvironment, highlighting a protein that was recently discovered to potently attenuate pancreatic cancer progression by influencing its cross-talk with the microenvironment.

**Abstract:**

Pancreatic cancer (PaC) is a highly aggressive disease, with poor response to current treatments and 5-year survival rates of 10–15%. PaC progression is facilitated by its interaction with the complex and multifaceted tumor microenvironment (TME). In the TME, cancer cells and surrounding stromal cells constantly communicate with each other via the secretion and uptake of factors including cytokines, chemokines, growth factors, metabolites, and extracellular vesicles (EVs), reshaping the landscape of PaC. Recent studies demonstrated that the metastasis suppressor N-myc downstream regulated 1 (NDRG1) not only inhibits oncogenic signaling pathways in PaC cells but also alters the communication between PaC cells and the surrounding stroma. In fact, NDRG1 was found to influence the secretome of PaC cells, alter cancer cell metabolism, and interfere with intracellular trafficking and intercellular communication between PaC cells and surrounding fibroblasts. This review will present recent advancements in understanding the role of NDRG1 in PaC progression, with a focus on how this molecule influences PaC-stroma communication and its potential for re-shaping the PaC TME.

## 1. Introduction

Pancreatic cancer (PaC) is known for its intractable malignancy, poor prognosis, and high fatality rates. In Australia, it is the deadliest cancer, with a five-year survival rate of just above 10% [1]. PaC was also identified as the seventh global cause of cancer-related deaths in 2020 [2,3]. Approximately 90% of exocrine PaC are classified as pancreatic ductal adenocarcinoma (PDAC) [4]. The most recent therapeutic recommendation for PaC patients with good performance status includes gemcitabine plus nab-paclitaxel (GEM/NAB-P), or a combination of fulminic acid, 5-fluorouracil, irinotecan, and oxaliplatin, also known as FOLFIRINOX [5]. However, these first-line regimens can induce severe adverse effects and promote tumor microenvironment (TME) transformation in favor of immunosuppression [6]. Current PaC treatments fail to significantly extend survival due to limited drug penetration into the hypovascularized and dense PaC TME.

**Figure 1:**
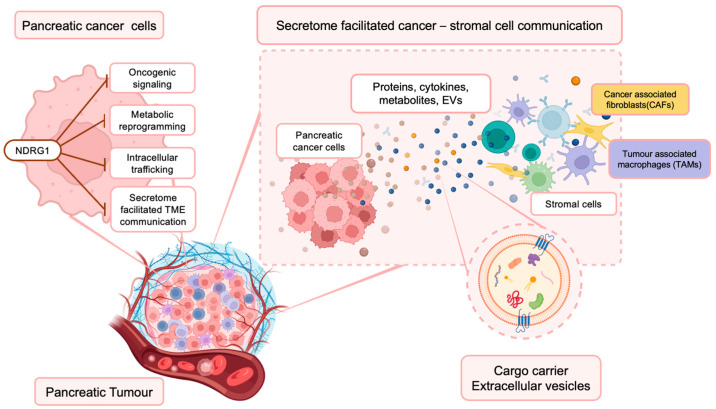
NDRG1 inhibits multiple oncogenic signaling pathways within PaC cells, influencing their secretome. This has important implications for the PaC TME, where cancer and surrounding stromal cells (i.e., cancer-associated fibroblasts (CAFs) and tumor-associated macrophages (TAMs)) communicate and influence each other’s function via the secretion of proteins, cytokines, metabolites, and extracellular vesicles that contain various bioactive materials.

**Figure 2:**
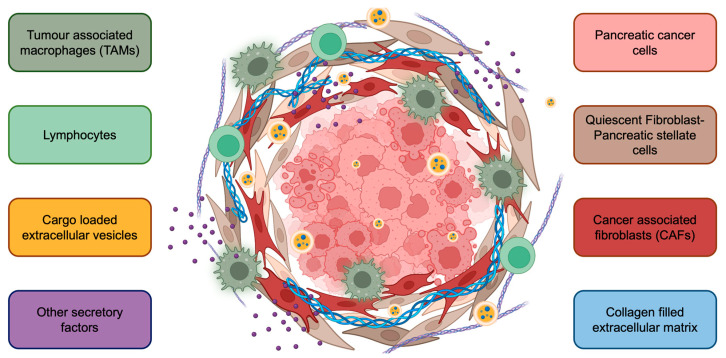
The complex tumor microenvironment of pancreatic cancer. Cancer cells are surrounded by stromal cells, including pancreatic stellate cells, cancer-associated fibroblasts, tumor-associated macrophages, and lymphocytes. Further, extracellular vesicles, various cytokines/chemokines, and the extracellular matrix contribute to PaC progression. The PaC TME also creates a physical barrier for drug penetration, contributing to limited response to therapy.

The PaC TME has been studied extensively over the past decade, with many studies demonstrating its multifaceted role in facilitating tumor growth and metastasis [7,8]. The stroma of PaC contains many cell types, including cancer-associated fibroblasts (CAFs), tumor-associated macrophages (TAMs), and other immune cells (i.e., regulatory T cells (Tregs), myeloid-derived suppressor cells (MDSCs), etc.) [9,10] (Figure 1). Another major component of the PaC TME is the dense extracellular matrix (ECM). The ECM is predominantly produced and maintained by CAFs [11]. These fibroblast-like cells support and protect PaC by building a dense collagen barrier around the cancer cells, consequently providing a favorable environment for tumorigenesis [12]. The TME of PaC was also found to be the major contributor to chemoresistance and is directly linked with poor clinical outcomes [6,13]. This is also facilitated by various cytokines, interleukins, growth factors, and extracellular vesicles (EVs) that are produced by both PaC and stromal cells and which act to establish a more immunosuppressive environment (Figure 2) [14]. Over the past decade, there has been an influx of studies that demonstrate tumor-secreted EVs in the cancer TME play a crucial role as communal messengers in early malignancy and during metastasis of cancer.

Recent evidence has demonstrated that the protein N-myc downstream regulated 1 (NDRG1) plays an important role in tumorigenesis and functions as a metastasis suppressor [15,16,17,18,19]. The downregulation of NDRG1 is associated with enhanced organ-specific tumor growth in breast [20], colon [21], prostate [22], cervical [23], ovarian [24], and pancreatic tissues [25,26,27,28]. More importantly, stable transfection or drug-induced upregulation of NDRG1 expression in PaC cells has been shown to prevent PaC metastasis formation [29,30]. The anti-cancer activity of NDRG1 is believed to be associated with its ability to regulate multiple oncogenic signaling pathways and alter cancer cell metabolism [31,32,33,34]. More recently, it was discovered that NDRG1 also influences the PaC secretome and subsequent cross-talk with the TME, leading to disrupted cancer-stromal cell communication [29,30] (Figure 1).

It has been well documented that NDRG1 can disrupt TGF-β-induced epithelial-to-mesenchymal transition (EMT) in cancer cells [31,35,36]. In the colon, prostate, and pancreatic cancer cells, overexpression of NDRG1 restored the co-localization of E-cadherin and β-catenin at the cell membrane [31,35,36], which facilitates the formation of the adherens complex [37]. More recently, Menezes et al. found that the increased expression of E-cadherin in response to NDRG1 overexpression was the result of reduced expression of E-cadherin transcriptional repressors, namely SNAIL, SLUG, and ZEB1 [35,36]. They also found that NDRG1 inhibits the oncogenic nuclear factor-κB (NF-κB) signaling pathway at various steps by reducing the expression levels of NEMO, Iĸĸα, and IĸBα. This study also identified that Iĸĸα, which integrates NF-κB and TGF-β signaling to upregulate ZEB1, SNAIL, and SLUG, is an NDRG1 target [35,36], which facilitates the formation of the adherens complex [37].

Moreover, NDRG1 was shown to regulate multiple other pro-tumorigenic pathways in PaC, including the phosphoinositide 3-kinase/protein kinase B (PI3K/AKT) and RAS pathways [38,39]. Upregulation of NDRG1 in prostate and PaC cells increased the expression of the tensin homolog deleted on chromosome 10 (PTEN), a negative regulator of the PI3K/AKT pathway [40]. NDRG1 also suppressed the RAS-RAF-MEK-ERK cascade by increasing the expression levels of SMAD4, another well-studied tumor suppressor [39,41]. In addition, NDRG1 was found to promote autophagy in PaC, as well as lysosomal-mediated down-regulation of the epidermal growth factor receptor (EGFR) [25,42].

While the majority of studies focused on the role of NDRG1 in various oncogenic signaling pathways in cancer, more recent work has revealed that this metastasis suppressor also influences metabolic reprogramming of cancer cells, disrupts lipid synthesis, regulates intracellular trafficking and degradation mechanisms, and impacts the TME. Intriguingly, NDRG1 has also been suggested to influence endosomal sorting and EV biogenesis [43]. Herein, we review the potential mechanisms by which NDRG1 might be regulating communication between PaC cancer cells and different components of the TME, providing potential new therapeutic opportunities to re-shape the PaC TME and enhance the efficacy of current treatments.

## 2. NDRG1

NDRG1 was first discovered in 1997 as a protein involved in the proliferation and differentiation of colon epithelial cells [44]. In normal cells, NDRG1 regulates multiple cellular pathways and processes relating to differentiation [44,45], lipid synthesis [46,47], Schwann cell myelination [48], and apoptosis [49]. The crystal structure of NDRG1 was only recently resolved [50], revealing that the functional core of NDRG1 consists of an α/β hydrolase domain and a CAP domain, which covers the protein binding site. This discovery suggests that upon its interaction with effector proteins, the CAP domain of NDRG1 undergoes a conformational change, allowing the binding and potential hydrolysis of substrates [51,52].

The NDRG1 protein is commonly expressed in the cytoplasm and nuclei of epithelial tissues and functions as a metastasis suppressor, particularly in pancreatic, prostate, and colon cancers [53,54]. However, a number of studies have also demonstrated a pro-tumourigenic role for NDRG1, particularly in hepatocellular carcinoma [55,56]. The role of NDRG1 in breast cancer remains controversial, with some studies showing a tumor-suppressive effect [57,58,59], while others suggest that NDRG1 promotes breast cancer progression [60,61]. For instance, high expression of NDRG1 in breast cancer cells was found to increase brain metastasis formation and was correlated with worse clinical outcomes and reduced survival [47,60]. Conversely, NDRG1 expression directly suppressed breast cancer bone metastasis in vivo, and was found to be correlated with improved relapse-free survival in Wnt-negative patient specimens [59].

These contrasting results suggest that the function of NDRG1 is heavily context-dependent, and further research is required to elucidate how its function is influenced in different cancer types and under different physiological conditions. For an in-depth review of NDRG1, its structure, and functions in cancer and other diseases, we refer the reader to excellent recent reviews on this protein [32,54,62,63,64].

## 3. Cancer Associated Fibroblasts and Desmoplasia

Desmoplasia is the production and proliferation of excessive fibrotic tissue surrounding cancer cells. Pancreatic desmoplasia was found to occupy up to 90% of the total tumor mass in rodents [65] and is one of the major histopathological features of PaC. The desmoplastic reaction leads to complicated and dysfunctional circuitous blood vessel networks, and high interstitial fluid pressure, leading to poor blood perfusion and a hypoxic, nutrient-depleted environment [66]. PaC cells survive in this hostile environment by forming a parasitic relationship with neighboring fibroblasts, relying on growth factors and metabolites produced by these stromal cells [67]. This section will outline the different fibroblasts found in the PaC TME and how NDRG1 influences their function.

### 3.1. Pancreatic Stellate Cells

**Figure 3:**
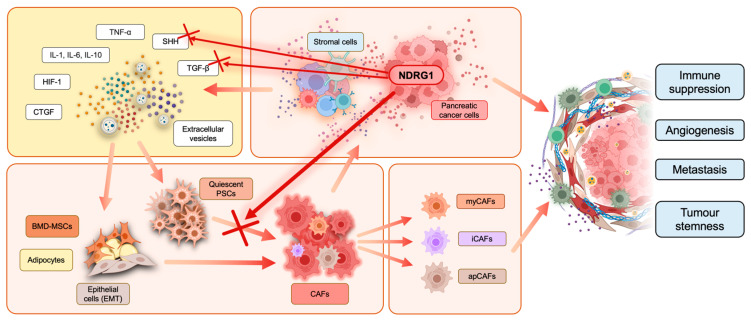
Cancer-associated fibroblast (CAF) activation in the PaC tumor microenvironment. Stromal and cancer cells release interleukins, tumor necrosis factor α (TNF-α), sonic hedgehog (SHH), transforming growth factor-β (TGF-β), hypoxia-inducible factor-1 (HIF-1), connective tissue growth factor (CTGF), and extracellular vesicles into the TME. These factors can interact with quiescent pancreatic stellate cells (PSCs), bone marrow-derived mesenchymal stem cells (BMD-MSCs), adipocytes, and epithelial cells, which undergo epithelial-to-mesenchymal transition (EMT). Moreover, this interaction promotes and induces cancer-associated fibroblast (CAF) transformation. There are three subtypes of CAFs often found in pancreatic cancer, including myofibroblastic CAFs (myCAFs), inflammatory CAFs (iCAFs), and antigen-presenting CAFs (apCAFs). CAFs release cancer-promoting factors into their surroundings to facilitate tumor progression. CAF activation and cross-talk with other cells in the TME result in overall immune suppression, tumor stemness, enhanced angiogenesis, and metastasis. Overexpression of NDRG1 in pancreatic cancer cells was recently reported to inhibit PSC activation in CAFs, as indicated by the red cross, potentially by interfering with the release of SHH and TGF-β from cancer cells.

Pancreatic stellate cells (PSCs) are myofibroblast-like star-shaped cells that can be found in the basolateral aspect of acinar cells or surrounding blood vessels and ductal epithelial cells in the functional pancreas [68]. In the normal pancreas, PSCs store vitamin A-rich lipid droplets, secrete hormones and cytokines supporting exocrine and endocrine homeostasis and immune surveillance, maintain a balanced composition of the stroma, and regulate ECM production [69]. Up until recently, PSCs were considered to be the major source of CAFs in PaC [70]. In fact, PSCs can transform into activated PSCs (aPSCs) in response to internal or external stimuli such as smoking, alcohol consumption, and hypoxia-induced stress, as well as in response to cell-secreted factors including various interleukins, TNF-a, SHH, HIF-1, TGF-β, connective tissue growth factor (CTGF), and EVs [71,72]. As a result of these pathophysiological changes, aPSCs begin to express high levels of α-smooth muscle actin (α-SMA), produce excessive ECM, and secrete growth factors that facilitate PaC metastatic progression [73] (Figure 3). In fact, high expression levels of α-SMA were demonstrated to correlate with poorer survival and therapeutic outcomes in PaC patients [73].

Hessmann et al. showed that aPSCs are directly involved in the chemoresistance of PaC by physically entrapping gemcitabine and consequently limiting drug delivery to cancer cells [74]. Several studies found that degradation of the abundant ECM component hyaluronan, which is produced by aPSCs [75], enhanced drug delivery and response to gemcitabine treatment using a mouse model [74,76,77]. PSCs are also believed to be the energy source for the oncogenic metabolism of PaC when nutrients in the TME are depleted [78]. Recent studies demonstrated that glutamine and other essential amino acids are significantly decreased in the pancreatic tumor core region compared to adjacent tissue [79,80]. To overcome this, PaC cells were found to enhance glutamine synthesis in neighboring PSCs by activating WNT-mediated β-catenin/TCF7 signaling in PSCs, leading to increased glutamine production and secretion into the TME [81]. This PSC-derived glutamine was vital in supporting the proliferation and survival of PaC cells under nutrient-depleted conditions [81]. Further, blocking glutamine uptake by PaC cells significantly reduced tumor proliferation in an orthotopic mouse model [81]. Another study suggested that PaC cells can induce programmed apoptosis in PSCs in order to gain access to alanine, which is an alternative fuel to support PaC growth [82]. Hence, accumulating evidence has confirmed the critical role that PSCs play in PaC development and chemoresistance.

### 3.2. Cancer-Associated Fibroblasts

Being a major constituent of the PaC tumor mass, CAFs secrete oncogenic factors that induce cancer angiogenesis and metastasis, increase stemness, and create an immunosuppressive TME [83,84,85,86,87]. CAFs are derived from resident fibroblasts [88], bone marrow-derived mesenchymal stem cells (BMD-MSCs) [89], adipocytes [90], aPSCs [91], and epithelial cells that have undergone EMT [92]. Neighboring non-malignant cells are transformed into CAFs following their exposure to cancer cell-derived factors, including SHH, TGF-β, and various interleukins [93,94,95]. Once activated, CAFs express various cell surface markers such as fibroblast activation protein (FAP) and α-SMA [96,97]. However, these biomarkers are not all-inclusive or CAF-specific, making it difficult to selectively manipulate CAFs using therapeutic strategies. In fact, Özdemir et al. demonstrated that non-specific targeting of CAFs accelerates disease progression and results in worse outcomes [98,99,100]. This has prompted a re-evaluation of the importance of stromal elements in PaC development and suggested that CAFs exist as a heterogenous population comprising both tumor-promoting and tumor-suppressing subtypes.

A breakthrough in our understanding of CAF heterogeneity came in 2017 when Öhlund et al. characterized two functionally and spatially distinct subpopulations of CAFs (Figure 3). These included myofibroblastic CAFs (myCAFs) and inflammatory CAFs (iCAFs) [70,101]. iCAFs were found to be distributed around the edges of a tumor away from cancer cells and produce inflammatory cytokines such as IL-6, with low expression of α-SMA [70]. Moreover, IL-1 was recently found to induce the transformation of aPSC into iCAFs through activation of the JAK/STAT pathway [102]. In contrast, myCAFs were proximal to cancer cells, produced TGF-β, and expressed high α-SMA levels [70]. Subsequent studies revealed a third CAF population, known as antigen-presenting CAFs (apCAFs) [101]. apCAFs express MHCII molecules, which were found to induce anergy or differentiation of CD4^+^ T-cells into immunosuppressive regulatory T-cells (Tregs) [101]. Notably, the apCAF population was recently found to be derived from mesothelial cells [103]. Recent studies have also revealed that different CAF populations exhibit metabolic heterogeneity, with glycolysis, fatty acid, and amino acid metabolism being highly active in iCAFs, while mitochondrial metabolism is more active in myCAFs [104].

Although each subtype presents with distinct characteristics, there is no single biomarker that is exclusively expressed by one subtype, and multiple markers are used to distinguish different CAF sub-types. It is possible that “hybrid” CAFs, possessing overlapping characteristics, also exist in PaC [105]. In fact, CAFs are highly plastic and can transform into different phenotypes depending on environmental cues [101,102]. Thus, it remains difficult to isolate and target specific CAFs. However, interestingly, a higher abundance of iCAFs was found to correlate with better prognosis, whereas a higher myCAF abundance correlated with worse prognosis in PaC, which may offer insight into the selective modulation of CAF subtypes [104].

### 3.3. NDRG1 Attenuates PSC Activation into CAFs

Recently, studies reported that NDRG1 can suppress tumorigenesis by limiting and regulating the cancer TME. In PaC cell models, Geleta et al. demonstrated that upregulation of NDRG1 can suppress the formation of desmoplasia by disrupting the communication between PSCs and PaC cells. Up-regulation of NDRG1 using the novel clinically trialed anti-cancer agent DpC effectively inhibited PaC growth and metastasis in vivo, while also reducing activation of PSCs and inhibiting ECM production [29,30]. Notably, this approach did not reduce the total number of PSCs in the tumor, as levels of the fibroblast marker, glial fibrillary acidic protein (GFAP), remained unchanged. Rather, up-regulation of NDRG1 by DpC attenuated the number of activated PSCs, as reflected by the reduced α-SMA levels and collagen deposition within the tumors [29]. However, the mechanisms by which NDRG1 inhibits PaC-PSC communication remain to be elucidated.

One potential mechanism by which NDRG1 inhibits cross-talk between PaC cells and PSCs/CAFs may involve its ability to suppress the production of secreted ligands from PaC cells. For instance, NDRG1 was found to significantly reduce the production of both SHH and TGF-β ligands by PaC cells [29] (Figure 3). As both SHH and TGF-β are key ligands involved in activating surrounding PSCs into CAFs [93], these results clearly demonstrate the impact NDRG1 has on the PaC TME. In fact, the effect of NDRG1 on reducing cancer cell TGF-β secretion may lead to fewer myCAFs in the TME, as this CAF sub-type is dependent upon TGF-β signaling [51].

Further studies by the same group also found that NDRG1 expression in PaC cells reduced their sensitivity to the proliferative effects of PSC-conditioned media, leading to reduced expression of tenascin C (TnC), β-catenin, cyclin D1, and c-myc proteins, which all promote PaC progression [30]. Moreover, PSCs exposed to conditioned media from PaC cells expressing high NDRG1 had significantly lower levels of α-SMA, β-catenin, TnC, and YAP/TAZ levels when compared to PSCs exposed to PaC cells with low NDRG1 expression [30]. The reduced β-catenin levels in these “NDRG1 conditioned” PSCs may impair their ability to produce glutamine, a vital mechanism by which they fuel PaC proliferation and which is dependent on activation of the β-catenin/TCF7 complex (see Section 3.1 above, [81]). Thus, NDRG1 is directly able to influence the PaC secretome to reduce PSC activation and potentially disrupt metabolic cross-talk.

Overall, this demonstrates the intriguing effect of NDRG1 on the bi-directional cross-talk between PaC and PSC cells, with NDRG1 expression in cancer cells likely reducing their ability to activate neighboring PSCs, which in turn produce fewer tumor-promoting factors to feed PaC progression. Future studies investigating whether NDRG1 might differentially influence the different CAF sub-types, and the functional consequences in terms of tumor progression are required to further establish the therapeutic potential of this metastasis suppressor.

## 4. Metabolic Cross-Talk in the TME

### 4.1. Role of Metabolic Reprogramming in PaC

**Figure 4:**
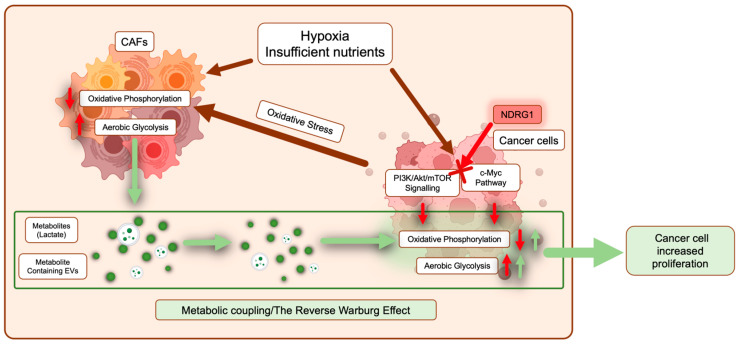
Pancreatic cancer cells are metabolically coupled with cancer-associated fibroblasts (CAFs). In response to a hypoxic and nutrient-deprived environment, both cancer cells and CAFs undergo metabolic reprogramming where normal energy production through oxidative phosphorylation is reduced, and aerobic glycolysis is promoted (red arrows). In cancer cells, major pathways involved in this reprogramming include PI3K/Akt/mTOR signaling and the c-Myc pathway. Moreover, cancer cells can promote metabolic reprogramming of CAFs by inducing oxidative stress (dark red arrows). As a result, CAFs release metabolites such as lactate and extracellular vesicles containing various metabolites, TCA intermediates, and amino acids, which are taken up by surrounding cancer cells to fuel both energy and biomass production (green arrows). NDRG1 inhibits PI3K/Akt/mTOR signaling and the c-Myc pathway (see red cross), potentially interfering with metabolic reprogramming in cancer cells.

Metabolic reprogramming of tumor cells is one of the hallmarks of PaC biology and contributes to chemoresistance [106]. Given the extensive metabolic cross-talk between PaC cells and the tumor microenvironment, researchers have increasingly focused on the therapeutic targeting of metabolic pathways to overcome the development of chemoresistance [107,108,109]. In contrast to normal cells, which produce the majority of their energy through mitochondrial oxidative phosphorylation, cancer cells favor aerobic glycolysis, known as the Warburg effect [110], as it supports more biomass production (Figure 4). Although this is a less efficient method of energy production, glycolytic cells produce key metabolites (i.e., pyruvate, lactate), amino acids (i.e., glutamine, alanine), and lipids to enhance proliferation, survival, and invasion [111,112]. In normal cells, pyruvate molecules produced by glycolysis are converted to acetyl CoA for entry into the tricarboxylic acid (TCA) cycle [113]. However, in cancer cells, pyruvate is instead converted into lactate, a process facilitated by lactate dehydrogenase A (LDHA) [114,115].

PaC metabolic reprogramming is underpinned by mutations in oncogenes such as KRAS [116], c-Myc [117], and the transcription factor HIF-1 [113]. Besides its well-known role in tumorigenesis and metastasis, mutated KRAS is also one of the most studied genetic signatures of PaC metabolism [118] and is even suggested to induce mitochondrial dysfunction [119,120]. The PI3K/Akt/mTOR (mechanistic target of rapamycin kinase) pathway is a downstream effector of KRAS activation [121] and is also involved in glucose metabolism in cancer cells [122] (Figure 4). PI3K is activated by upstream signaling from a variety of receptor tyrosine kinases (RTKs) and G protein-coupled receptors (GPCRs) [121]. Active PI3Ks phosphorylate membrane phospholipid PIP_2_ to PIP_3_, which facilitates phosphorylation and subsequent activation of AKT [121]. Consequently, AKT activation increases glucose uptake, glycolytic rate, and lactate production [121,123] by regulating post-transcriptional modifications of glycolytic enzymes such as hexokinase 2 (HK2) and 3-phosphoinositide-dependent kinase 1 (PDK1) [122].

Another key regulator of PaC metabolism is the c-Myc protein, which is encoded by the MYC proto-oncogene [124] (Figure 4). MYC and HIF-1 collaboratively upregulate multiple genes in favor of aerobic glycolysis. These genes include glucose transporter (GLUT1), HK2, pyruvate kinase M2 (PKM2), LDHA, and PDK1 [125]. This switch enables cancer cells to produce key substrates that fuel tumor growth, invasion, and metastasis under oxygen- and nutrient-starved conditions [126].

CAFs are also implicated in PaC metabolic re-programming, being recruited by the cancer cells to produce metabolites that are then scavenged by PaC cells. Cancer cells induce oxidative stress in neighboring CAFs, causing the aforementioned glycolytic switch [127]. In turn, glycolytic CAFs similarly produce energy-rich metabolites and TCA intermediates to fuel tumor growth under oxygen- and nutrient-depleted conditions [111,127]. CAFs and cancer cells interact through various signaling pathways to facilitate the exchange of metabolic substrates, and this is known as the reverse Warburg effect [127,128] (Figure 4). Under hypoxic and nutrient-depleted conditions, CAFs increase the packaging and release of EVs, which are subsequently taken up by cancer cells to fuel their metabolism [129] (Figure 4). Monocarboxylate transporters (MCTs) are overexpressed in both cancer cells and CAFs to transport lactate and maintain intracellular pH balance [86,130,131]. Interestingly, neighboring normoxic cells also express MCTs and take up lactate from hypoxic PaC cells, facilitating their entry into glycolysis and further promoting the proliferation of the cancer cells [132,133]. Production of glutamine, a critical nutrient required for PaC cell proliferation, is also promoted by aPSC-derived CAFs via the upregulation of glutamine anabolic pathway genes, such as glutamine synthetase [81].

Cancer cells can adapt to adverse conditions created by chemotherapeutics by leveraging CAF metabolic outputs [106,127]. These metabolic changes have an overall immunosuppressive effect as the different TME components compete for glucose, decreasing the availability of glucose for infiltrating T-cells, which hinders their activation and tumor-suppressive effects [111,134]. Resulting from the increased glycolysis, the accumulation of lactate in the TME is also important in facilitating extracellular acidosis, which inhibits natural killer (NK) cell activity by downregulating the expression of the natural cytotoxicity receptor NKp46 and increasing MDSC expression [135]. Moreover, weakly acidic and alkaline chemotherapies are neutralized in a highly acidic PaC TME [136]. Enhanced glycolysis and lactate accumulation take place alongside de novo lipogenesis as well as HIF-1-mediated immunosuppression. High expression of fatty acid synthase (FASN), a key enzyme in lipogenesis, has been found to correlate with poor patient prognosis and gemcitabine resistance in various human PaC cell lines [137]. FASN facilitates chemoresistance by mediating endoplasmic reticulum (ER) stress [137] and upregulating PKM2 expression [138]. Interestingly, in a study conducted by Tadros et al., drug resistance was only caused by fatty acid biosynthesis, not extracellular fatty acid uptake [137].

As a result of inefficient angiogenesis, regions of hypoxia develop, and this is believed to be a crucial factor that contributes to more aggressive disease progression and poor prognosis [139]. Hypoxic tumor cells are protected from apoptosis as anaerobic glycolysis becomes the preferred method for energy production in response to insufficient oxygen perfusion [140]. In PaC, hypoxia is a strong activator of HIF-1α [122], with HIF-1α being stabilized by the PI3K/Akt pathway [141]. Activated HIF-1α then drives the switch to glycolysis exhibited by PaC cells [142]. Moreover, HIF-1α signaling is involved in the recruitment and differentiation of MDSCs into tumor-promoting M2 TAMs [143] as well as the recruitment of immunosuppressive Tregs into the TME [144]. In line with these findings, suppressing glucose metabolism and HIF-1α signaling has been found to improve tumor sensitivity to chemotherapies [145,146].

The immature and leaky blood vessels within tumors, combined with extensive fibrotic ECM, manifest a perfect defense system against the delivery of anti-cancer therapeutics [147,148]. Conway et al. discovered that hypoxia moves transiently around the PaC TME, suggesting that the drug resistance associated with hypoxia and subsequent desmoplasia in these regions similarly transits around the tumor [149]. Desmoplasia disrupts the balanced communication between cells and regulatory components, ultimately contributing to tumor progression and the development of resistance to current therapies.

### 4.2. NDRG1 Is Involved in PaC Metabolic Reprogramming and Lipid Metabolism

NDRG1 regulates critical metabolic regulators, including MYC, PI3K/AKT, and HIF-1α. In breast cancer cells, upon reoxygenation, NDRG1 expression is significantly reduced while c-Myc protein expression is upregulated [150]. It has been well established that NDRG1 is downregulated by both N-Myc and c-Myc [46], potentially via binding to and methylation of the NDRG1 promotor region [32]. Another group similarly reported that c-Myc is associated with the E2F-MYC activator, MYC-associated zinc fingers, and E-box-binding factors in the NDRG1 promoter region [150]. Furthermore, NDRG1 has been shown to inhibit the PI3K pathway by reducing the expression of key subunits, including phosphorylated-p85α (p-p85α) and phosphorylated-p55𝑦 (p-p55𝑦) [151]. This may be a downstream effect of NDRG1’s ability to increase the expression of PTEN, which acts as a negative regulator of PI3K signaling [51,52]. One team found that overexpression of NDRG1 in PaC cells can result in modulated aerobic glycolysis by regulating GLUT1, HK2, LDHA, and PDK1 expression [34]. These results also suggest that NDRG1 can suppress the activity of HIF-1α [34]. In line with this, Liu et al. suggested that HIF-1α suppression could be a downstream target of NDRG1 in a PI3K/Akt signaling-dependent manner [51,52] (Figure 4).

Early evidence suggests that NDRG1 may also regulate lipid metabolism. Yeast two-hybrid screening demonstrated that NDRG1 interacts with high-density lipoproteins, apolipoproteins A-I and A-II [151]. Another study examining breast cancer found that silencing NDRG1 caused dysregulated lipid metabolism, leading to increased fatty acid conversion to neutral lipids and lipid droplets [47]. In this study, NDRG1 was found to be an important regulator of lipid fate in breast cancer, where it was also associated with poor prognosis [47]. More recently, the crystal-soluble structure of NDRG1 has been resolved, which suggests that the core functional domain of NDRG1, which is involved in its direct binding to other proteins, constitutes an α/β hydrolase fold [51,52]. Lipase is another member of the α/β hydrolase superfamily [52] with its active site being the α/β hydrolase fold, hidden under a secondary structure, the CAP domain, which is similar to the structure of NDRG1 [51,52]. Based on this, it may be speculated that NDRG1 potentially functions as a lipase, although this remains to be investigated.

Overall, NDRG1 can regulate key molecular pathways that influence cancer cell metabolism. However, whether NDRG1 expression in PaC cells can also influence the metabolic reprogramming of other cells in the TME such as CAFs or immune cells remains to be elucidated.

## 5. Immunomodulation in the PaC TME

In addition to desmoplasia, another major TME characteristic of PaC is an immunosuppressive microenvironment. In fact, PaC patients usually present with intense immune dysfunction accompanied by unregulated production of proinflammatory cytokines and an abundance of immunosuppressor cells [152].

### 5.1. Roles of Suppressor T Cells, Myeloid-Derived Suppressor Cells and Tumour Associated Macrophages

Suppressor T cells, including CD4^+^, CD25^+^, and Foxp3^+^, also known as Tregs, are part of the subtype of T cells that prevent autoimmune diseases and inflammatory disorders [153]. Tregs were found to be responsible for the inhibition of antitumor immunity in murine models via the TGF-β signaling pathway [154]. Many studies have established that there is an accumulation of Tregs in human and mouse pancreatic cancer [155,156,157]. More importantly, high levels of Tregs were correlated with increased PaC metastasis and poor prognosis in patients [158].

MDSCs are a heterogeneous population of immature myeloid cells (IMCs) and are associated with many diseases such as obesity, autoimmune abnormalities, chronic inflammation, and pancreatic cancer progression [159,160]. The level of MDSCs in cancer has been elucidated to not only suppress the natural antitumoral activities of the host immune system but also promote oncogenic cellular pathways that favor tumor growth, angiogenesis, and metastasis [161]. Blood collected from PaC patients was found to have a high frequency of MDSCs and pro-MDSC cytokines such as IL-6 [160]. Depletion of myeloid cells was found to prevent the initiation of KRAS-driven pancreatic tumorigenesis by downregulating the secretion of programmed cell death ligand 1 (PD-L1), which restored the anti-tumor activities of CD8+ T cells [161]. Another mechanism by which MDSCs enable immune evasion is by releasing reactive oxygen species (ROS) [162], which induce oxidative stress in T cells and reduce their proliferation [162].

Macrophages are also an important component of the pancreatic TME and are derived from monocytes. Monocytes differentiate and polarize into M1 or M2 macrophages in response to different stimuli [163]. M1 macrophages are activated by IFN-γ and TLR ligands and produce high levels of IL-12, IL-23, MHC II, and iNOS [164]. Their main function is to identify and prevent cancer initiation. In contrast, M2 macrophages, also known as tumor-associated macrophages (TAMs), are induced by IL-4, IL-10, IL-13, and TGF-β, and can facilitate cancer growth [164]. TAMs are believed to contribute to stromal remodeling and immunosuppression in PaC development. Studies showed that M2 macrophages are the predominant macrophage phenotype in PaC and that their infiltration negatively impacts overall survival [165]. TAMs can also produce enzymes such as Arg1 to uptake exogenous L-arginine from the TME, which affects the metabolism of T cells and reduces their activation [166]. TAMs also release Treg recruiting factors such as TGF-β, IL-10, and prostaglandin E2 (PGE2) to further disrupt the cytotoxic CD8+ T cell population [167,168].

### 5.2. CAF Mediated Immune Suppression

Many proteins secreted by CAFs have demonstrated direct inhibitory effects on immune cells. For instance, CXC chemokine ligand 12 (CXCL12) released by aPSC/CAFs can restrict the infiltration and migration of CD8^+^ T-cells, preventing them from reaching cancer cells [169]. Moreover, aPSC/CAFs with high expression of Galectin-1 and α-SMA found in close proximity to tumor cells form a tight fibrotic barrier via ECM deposition, restricting blood flow and thus preventing immune cell infiltration [152]. Further, high expression of endogenous Galectin-1 by aPSCs induced apoptosis of CD4+ and CD8+ T cells [170]. CAFs were also found to recruit Tregs and reduce the activity of Th1 lymphocytes by upregulating interferon-γ inducible protein 10 (IP-10) [171]. In addition to this, CAFs can also hinder T cell proliferation by regulating the release of PGE2, TGF-β and vascular endothelial growth factors (VEGF) [172,173,174,175,176]. IL-6 secreted from iCAFs activates the signal transducer and activator of the transcription 3 (STAT3) pathway, which promotes differentiation of immune-suppressing MDSC cells [177]. Studies also suggested that the macrophage-aPSCs interaction is an essential part of desmoplasia formation in PaC, with TAMs enhancing ECM secretion by aPSCs [178,179].

### 5.3. Does NDRG1 Regulate Immune Cell Differentiation?

There is limited evidence suggesting NDRG1 can modulate immune cells in the context of cancer. NDRG1-deficient mice were found to have an imbalance in macrophage lineage cell differentiation, leading to pathological angiogenesis and suppressing bone remodeling [180]. Further, low levels of NDRG1 were correlated with an immature immune cell phenotype, and NDRG1 was found to be functionally involved in the maturation of neutrophils [181]. NDRG1 was also found to have a crucial role in the degranulation and terminal maturation of mast cells in mice [182]. In hepatocellular carcinoma, the expression of NDRG1 was positively correlated with T follicular helper, Th2, and NK cells and negatively correlated with plasmacytoid dendritic cells and Th17 cells [183]. In human gastric cancer cells, overexpression of NDRG1 was found to promote infiltration of TAMs and increased expression of the inflammatory cytokine IL-1α [184].

Overall, these studies demonstrate that NDRG1 may be an important factor influencing immune cell infiltration and activation. However, the effect of NDRG1 on immune cells in the PaC TME remains to be established. Considering that NDRG1 suppresses TGF-β secretion by PaC cells [29], which plays a central role in immunosuppression [185], it is tempting to postulate that this may enhance the immune response. This hypothesis is further supported by the NDRG1-mediated de-activation of CAFs, as these are other facilitators of immunosuppression (see Section 5.2). While the mechanisms by which NDRG1 influences the dynamic cross-talk between PaC cells, CAFs, and immune cells remain to be elucidated, recent evidence suggests that EVs may be involved, and this will be further discussed below.

## 6. Extracellular Vesicles Facilitated Cross-Talk in the TME

EVs are nanosized membrane-encapsulated vesicles loaded with bioactive material such as nucleic acids, fatty acids, proteins, and metabolites that are secreted by almost all cells [186]. There are two major types of EVs characterized by their size and distinctive biogenesis pathways, which include exosomes (30–150 nm) and microvesicles (MV; 100–1000 nm) [187]. EVs act as a cellular modulator that interacts with other cells in local and distant locations, potentially altering the activity of the receipt cells [188]. Moreover, EVs can facilitate cancer cell migration and invasion of surrounding and distant tissues [189]. Hence, EVs have emerged as an important target for cancer research [190].

EVs are released by cancer cells through different biogenesis pathways to interact with cells within the TME and create a favorable niche for proliferation and invasion [191]. These signals often promote oncogenesis [192], angiogenesis [193], EMT [194], ECM remodeling [195], and immunological tolerance [8,191]. Mounting evidence suggests that the oncogenic initiation of PaC is tightly associated with cellular alterations caused by EVs [8]. Importantly, recent studies also highlighted that PaC-derived exosomes carry abundant oncogenic and cancer-inducing factors such as microRNAs (miRNA) and proteins that can transform the TME and enhance cancer progression [196,197,198,199]. Numerous studies have also demonstrated that manipulation of the biogenesis pathways of EVs can lead to impaired exosome release and cargo sorting capacity [197,198,199].

### 6.1. EV Biogenesis

**Figure 5:**
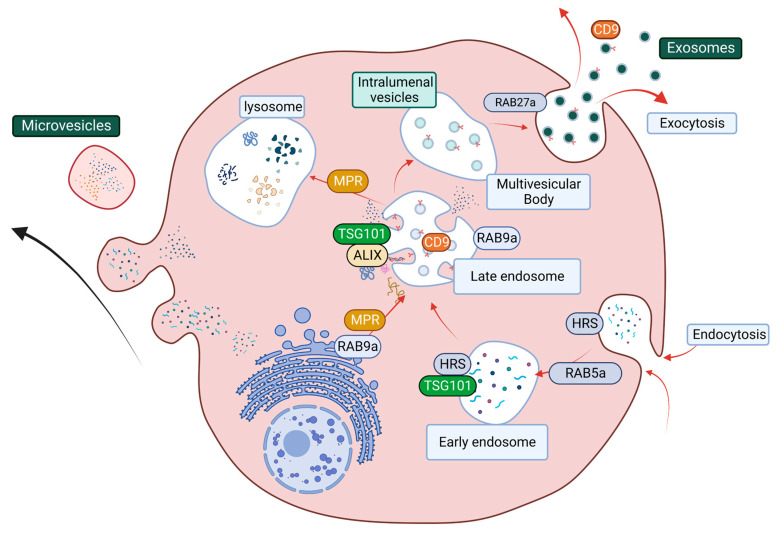
The structural network of EV biogenesis that is facilitated by the ESCRT pathway and Rab protein-related vesicle transportation and trafficking. The ESCRT pathway starts at endocytosis (Rab5a) with the maturation of early endosomes (HRS and TSG101). ESCRT accessory cargo sorting protein ALIX and Rab9a facilitate cargo sorting and late endosome maturation. Rab9a also promotes lysosome formation by transporting mannose-6-phosphate receptor (MPR) from the trans-Golgi network. MPR then transports digestion enzyme hydrolases from the late endosome to the lysosomes. The late endosome can mature into multivesicular bodies, and the packaged intraluminal vesicles then get released through the plasma membrane as exosomes.

Exosomes are the major type of EVs, and their biogenesis and release include a series of tightly regulated events. In the early stage of exosome biogenesis, endosomes first reach maturation, resulting in the formation of multivesicular bodies (MVBs), which contain intraluminal vesicles (ILVs) [200,201,202]. MVBs then fuse with the cell plasma membrane to release ILVs into the extracellular space as exosomes [200]. Alternatively, MVBs can also fuse with the lysosome, leading to the degradation of ILVs and their cargo [200,201,202]. This process involves two main regulatory complexes, the endosomal complex required for transport (ESCRT) pathway and the GTPase Rab family of proteins that facilitate vesicle trafficking (Figure 5; [200]). The ESCRT pathway predominately regulates both exosome biogenesis and cargo sorting and contains distinct sub-complexes that regulate different parts of exosome biogenesis, namely ESCRT-0 (HRS), ESCRT-I (TSG101), ESCRT-II, and ESCRT-III, as well as associated protein ALG-2 interacting protein X (ALIX) [203]. Rab family members, including Rab5a, Rab9a, and Rab27a, facilitate internal organelle maturation, formation, and fusion events [200,204,205] (Figure 5).

Numerous studies have demonstrated that manipulation of these pathways can lead to impaired exosome release and cargo sorting capacity [197,198,199]. Published work by Ostrowski et al.; Gorji-Bahri et al. has identified that reduced levels of either Rab27a, Rab5a, or Rab9a can lead to decreased exosome secretion in cancer cells [198,199]. However, whether manipulation of EV biogenesis pathways can result in altered EV release has never been examined in PaC. These discoveries have indicated that EVs are an important and valuable target for PaC, which require further attention. The cargo of PaC-derived EVs and their ability to influence disease progression will be discussed further below.

### 6.2. PaC EV Cargo

The molecular cargo of PaC EVs has been extensively explored by recent studies. EVs derived from both cancer and stromal cells have the ability to transform the TME in favor of cancer progression. CAF and PSC-derived EVs containing miRNA cargo were demonstrated to promote PaC cell proliferation, migration, and metastasis in both cell and animal models. Stromal EVs were found to contain miRNA-10a-5p (CAF derived), ANXA1 (PSC derived), miRNA-5703 (PSC derived), miRNA-222 (PSC derived), and LncRNA-SBF2-AS1 (TAM derived), with these components being demonstrated to promote tumor cell proliferation, migration, and metastasis in both cell and animal models [206,207,208,209,210]. Moreover, miRNA-106-5p (CAF derived) and miRNA-155 (PaC derived) can induce and enhance cancer cell gemcitabine resistance [211,212].

A recent study demonstrated that the transformation of fibroblasts into CAFs was attributed to PaC-derived exosomes, with exosomes derived from normal cells having no effect [213]. Further, PaC patients were found to have elevated exosome levels in their plasma when compared with healthy controls [214]. More importantly, reducing PaC cell EV secretion via microRNA interference can attenuate gemcitabine resistance [214]. Another team found that blocking PaC tumor cell release of EVs containing macrophage migration inhibitory factor (MIF) can prevent liver metastasis formation [215].

Due to the multifaceted role of EVs in PaC tumorigenesis, the cargo of PaC EVs is being increasingly explored for both early diagnostic and treatment development. One recent study isolated EVs from the plasma of PaC patients and identified several tumor-promoting markers being consistently upregulated in these EVs, including LAMA5, SDCBP, and TENA [216]. Another study demonstrated that MVs containing CD44, PPP2R1A, and TP53 purified from PaC patients can induce PANC-1 cells to obtain a metastatic phenotype with enhanced migration and proliferation [216,217]. Studies by Wang et al. discovered that EV-derived VEGF-C can induce PaC cell migration and metastasis by facilitating and promoting immune evasion and lymphangiogenesis [218,219]. Moreover, inhibition of VEGF-C packaging into EVs can potentially reverse PaC initiation [218,219]. However, the role of EVs in PaC and how their cargo is regulated remains to be characterized and holds great promise in the development of innovative therapeutic approaches as well as the potential early diagnosis of this aggressive disease.

### 6.3. EV Targeted Approaches

New approaches targeting the biosynthesis of EVs have recently been explored in PaC. It has been observed that the absence of Annexin A1 (ANXA1), a key promotor and regulator of PaC development and drug resistance [220,221,222], dramatically decreases the quantity of secreted exosomes by PaC cells [207]. Another known protein factor that promotes PaC cellular proliferation and survival is GAIP-interacting protein C terminus (GIPC) [223]. This protein is also involved in intracellular vesicle trafficking [224]. A recent study found that elevated GIPC in PaC cells results in increased secretion of exosomes by upregulating the expression of ALIX, TSG101, and charged multivesicular body protein 4B (CHMP4B) [224].

Overall, many studies have presented evidence highlighting the essential role of EVs and their molecular cargo in PaC development and chemoresistance. More importantly, inhibition of EV biogenesis can potentially disrupt communication between PaC and its TME, thus hindering tumor progression and metastasis. Recent studies have identified that the metastasis suppressor NDRG1 can potently inhibit the cross-talk between PaC cells and stromal PSCs [29,30]. Intriguingly, NDRG1 has also been suggested to influence endosomal sorting and EV biogenesis [43,225,226], although its effects on PaC EV secretion and EV cargo packaging have never been assessed. The function of NDRG1 and its links to EV biogenesis will be further discussed below.

### 6.4. NDRG1 Regulates Intracellular Trafficking

There is limited direct research indicating the potential impact of NDRG1 on the biogenesis of EVs. However, multiple studies have shown that NDRG1 might regulate intracellular vesicle trafficking via its direct or indirect interaction with the small GTPase Rab family of proteins [226]. NDRG1 can directly interact with the prenylated Rab acceptor 1 (PRA1) protein, which then regulates small GTPase Rab proteins that facilitate transport between cell organelles [151]. The inherent function of PRA1 is to promote vesicle formation and release from the Golgi complex, recruiting proteins that are necessary for cargo sorting and subsequent vesicle docking and fusion [227]. These include Rab7, which is essential for late endosomal vesicle trafficking [228].

Moreover, NDRG1 was found to directly bind to Rab4a to regulate E-cadherin transport and recycling through an endosome-related mechanism [226]. In addition, NDRG1-silenced cells were found to have impaired lysosomal function and increased secretion of neutral sphingolipids and ceramides [43,47,226], which are known to affect exosome biogenesis [229]. In fact, this latter study found that NDRG1-silenced cells had a significantly increased release of functional exosomes [43]. Hence, enhanced NDRG1 expression may potentially impact exosome biosynthesis and/or release, and this could be one of the mechanisms behind its ability to inhibit the PaC-TME cross-talk. Further studies are required to elucidate the effects of NDRG1 on EV biogenesis and cargo loading and may lead to promising new approaches to overcome PaC progression and metastasis.

## 7. Conclusions

Finding a reliable biomarker for early PaC diagnosis and developing better treatment strategies for this disease remains a significant challenge. For both early detection and therapeutic development, the primary challenge is to overcome the complex TME remodeling, where cancer cells constantly alter stromal cells for survival. As a result, the PaC TME undergoes pro-oncogenic transformation, giving rise to a metabolically active and immunosuppressive phenotype under hypoxic and nutrient-poor conditions. This eventually establishes an impenetrable desmoplastic barrier that impairs drug delivery and promotes the development of resistance. In this review, we have brought together evidence that suggests the metastasis suppressor NDRG1 plays an important and multifaceted role in the cross-talk between PaC cells and the TME. As a potent inhibitor of oncogenic signaling in PaC, NDRG1 was found to limit the production of cytokines and growth factors that influence the TME, consequently reducing tumor progression and inhibiting metastasis. Its effects on key proteins involved in cancer cell metabolism and its potential ability to affect immune cell differentiation further highlight its involvement in the TME. Finally, recent evidence also suggests that NDRG1 affects intracellular vesicle trafficking and may potentially influence exosome release. Elucidating the effects of NDRG1 on these different constituents of the PaC-TME cross-talk may lead to novel therapeutic approaches to overcome the oncogenic effects of the TME and enhance the efficacy of current PaC therapies.
